# Prediction of Adverse Maternal Outcomes in Women with Early-Onset Preeclampsia with Severe Features in Mexico: Validation of the Full-PIERS Model

**DOI:** 10.1155/2024/5520312

**Published:** 2024-05-15

**Authors:** Eduardo Ponce Nájera, Marcela Montoya Hinojosa, Rogelio Armando Lozano Galván, Diego González Oropeza

**Affiliations:** ^1^Department of Obstetrics, Hospital Regional Materno Infantil del Estado de Nuevo León, Monterrey 67140, Mexico; ^2^Resident of the Multicenter Program of Medical Specialties, Tecnológico de Monterrey and Servicios de Salud de Nuevo León, Nuevo Leon, Mexico; ^3^Escuela de Medicina y Ciencias de la Salud Tecnológico de Monterrey, Nuevo León, Mexico; ^4^Hospital Zambrano Hellion TecSalud, Monterrey 66260, Mexico; ^5^Multicenter Program of Medical Specialties, Tecnológico de Monterrey and Servicios de Salud de Nuevo León, Nuevo Leon, Mexico

## Abstract

**Objective:**

Validate the full-PIERS model in predicting adverse maternal outcomes in women with early-onset preeclampsia with severe features in our population.

**Methods:**

Retrospective cohort study. We applied the full-PIERS model on 130 women with severe early-onset preeclampsia who were treated at a second-level hospital in Nuevo León, México. We validated the full-PIERS model in its ability to discriminate through the AUROC.

**Results:**

The full-PIERS model applied to the data obtained in our study had good discrimination, revealing an AUC of 0.718 (95% CI 0.515–0.921; *P* = 0.017). A cut-off of 7.95 was identified as the cut-off point with the best diagnostic performance, with the highest Youden index, presenting a sensitivity of 54.5% and specificity of 99.2% for the development of complications.

**Conclusion:**

The full-PIERS model can predict adverse maternal outcomes in women admitted to our hospital with severe early-onset preeclampsia within 48 hours of admission.

## 1. Background

Hypertensive disorders associated with pregnancy can affect up to 5% of pregnancies worldwide, contributing significantly to both maternal and fetal morbidity and mortality [1]. Among these disorders is preeclampsia, which can be classified as early-onset preeclampsia (diagnosed before 34 weeks of gestation) or as late-onset preeclampsia (diagnosed after 34 weeks of gestation) [2]. Although late-onset preeclampsia is more prevalent, early-onset preeclampsia is associated with more severe maternal and neonatal outcomes [3, 4]. In Mexico, hypertensive disorders in pregnancy represent one of the leading causes of maternal death, and there is currently no tool that allows the prediction of adverse maternal outcomes in women with early-onset preeclampsia [5]. Evaluating the performance of the full-PIERS model in our population is of great importance to establish its accuracy and predictive capacity and thus establish its use in our hospital care centers as a viable and cost-effective alternative to help reduce maternal and neonatal morbidity and mortality.

## 2. Methods

A single-center retrospective cohort study was conducted on women with a single pregnancy, diagnosed with severe early-onset preeclampsia, without other comorbidities, who were treated in the period from January 1, 2021, to December 31, 2022, at the Department of Gynecology and Obstetrics in the “Hospital Regional Materno-Infantil” in Nuevo León, México and that had all the required data to input in the full-PIERS model.

Preeclampsia's severe features were defined as systolic pressure ≥160 mmHg or diastolic pressure ≥110 mmHg on two occasions more than 15 minutes apart, creatinine >1.1 mg/dL, thrombocytopenia (platelets < 100,000), progressive renal failure, decreased liver function, pulmonary edema, or signs of hypertensive encephalopathy [6]. The variables required to perform the calculation in the full-PIERS model were collected: weeks of gestation, serum concentrations of aspartate transaminase, oxygen saturation, platelet count, chest pain or dyspnea, and serum creatinine [7]. The presentation of maternal and perinatal complications within 48 hours of their arrival and hospitalization was also collected. For this study, the adverse maternal outcomes we included were obstetric hemorrhage, need for blood transfusion, HELLP syndrome (hemolysis, elevated liver enzymes, and decreased platelet count), thromboembolism, acute pulmonary edema, brain hemorrhage, liver or kidney failure, uncontrolled blood pressure, and fetal or maternal death.

The data obtained from the electronic files are entirely anonymous; a database was filled with exclusive access to the researchers.

This study was approved by the “Hospital Regional Materno-Infantil” ethics committee. It was carried out in full compliance with the ICH E6 guideline of Good Clinical Practices and with the principles of the Declaration of Helsinki.

The sample size calculation was calculated using a diagnostic test formula. Based on literature data indicating a negative predictive value of 96%, with a maximum allowable width of confidence interval set at 7% and a two-tailed significance level of 5%, a minimum of 121 patients was deemed necessary [7].

The data were subsequently analyzed to validate the prediction capacity of the full-PIERS model, and it was compared with the sensitivity and specificity data against the values reported in the development of the full-PIERS model. This study included elements of the STARD checklist guidelines for diagnostic test reporting studies [8]. A receiver operating characteristic (ROC) curve and the area under the curve (AUC) were obtained, along with a 95% confidence value, to validate the prediction capacity of the full-PIERS model. The following values were taken as reference: inconclusive (AUROC ≤ 0.5), poor discrimination (0.5 < AUROC < 0.7), and good discrimination (AUROC ≥ 0.7) [7, 9]. The cut-off point that reflects the best stratification power was selected.

In terms of categorical variables, this study utilizes frequency tables and cross tables to examine differences between groups of categories. For continuous variables, summaries of means or medians are presented along with their corresponding standard deviation or interquartile range, depending on their distribution. Sensitivity and specificity are illustrated on the ROC curve plot, with the AUC section highlighted to convey the model's predictive power and validate the primary endpoint in this population. The Youden index (IY) was calculated, and the cut-off point was isolated by searching for the highest Youden index to report the cut-off point with the best diagnostic performance. We used Pearson's chi-square test or Fisher's exact test to compare categorical variables, while numerical variables were compared using the Mann–Whitney test. IBM SPSS Statistics for Windows, Versión 24.0. Armonk, NY: IBM Corp programs were used.

## 3. Results

The present study analyzed 24936 deliveries within the 24 months considered. Preeclampsia with severe features was diagnosed in 742 patients (2.97%); of those, 283 patients were diagnosed with early-onset preeclampsia with severe features, corresponding to 1.1% of the total deliveries, and 38% of the patients with preeclampsia with severe features. For this study, 130 observations of women who presented with early-onset preeclampsia with severe features and met the inclusion criteria at the Gynecology and Obstetrics emergency department of the “Hospital Regional Materno-Infantil” in Nuevo León, México were included.

The median age of the women was 27 (21–34) years. Among the total women, 11 (8.5%) experienced complications. Tables 1–4 compare women who presented complications with those who did not. A more significant proportion of women with complications were found to be married (63.6% vs. 16%, *P*=0.001).

Prenatal control was adequate in 83 (63.8%) women, with no association with complications. Women who experienced complications had a lower parity (1 vs. 2 previous pregnancies, *P*=0.011), and those without complications had a higher history of caesarean section (42.9% vs. 9.1%, *P*=0.025). The median weeks of gestation between the groups were similar (Table 1).

No women presented dyspnea, and 3 (2.3%) developed chest pain. Regarding laboratory parameters and vital signs, we documented a higher median creatinine in women who experienced complications (0.73 vs. 0.6 mg/dL, *P*=0.005), as well as higher ALT (25 vs. 18 U/L, *P*=0.03), and a nonsignificant trend of more elevated AST (28 vs. 21 U/L, *P*=0.061). No differences were observed in blood pressure, platelet levels, oxygen saturation, or proteinuria (Table 2).

A total of 11 (8.5%) patients presented an adverse maternal outcome. Of those 11 patients, four patients presented HELLP syndrome, three patients presented with uncontrolled blood pressure, two patients presented stillbirth, one patient presented renal failure, and one patient presented obstetric hemorrhage. A higher full-PIERS score was documented in women who developed complications (18.7 vs. 0.5, *P*=0.017), along with a lower history of corticosteroid administration (36.4% vs. 84.9%, *P*=0.001), with no difference observed in the use of antihypertensives. All women received eclampsia prophylaxis with magnesium sulfate according to our hospital protocols (Table 4).

No difference was found in the median hospital stay or the days from admission to birth. Women with complications exhibited a higher stillbirth rate (18.2% vs. 1.7%, *P*=0.036). There were no differences in the newborns' weight or the proportion of pregnancy resolution pathways (Table 4).

**Figure 1:**
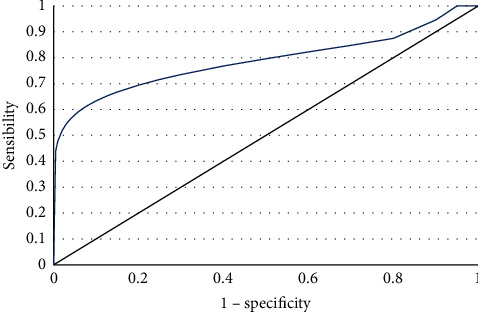
ROC curve of full-PIERS performance for the development of complications.

An analysis using ROC curves was performed to assess the predictive performance of the full-PIERS score for the development of complications (Figure 1), documenting an area under the curve (AUC) of 0.718 (95% CI 0.515–0.921; *P*=0.017). Table 4 presents the full-PIERS cut-offs along with the sensitivity and specificity of each one. A cut-off of 7.95 was identified as the point with the best diagnostic performance, having the highest Youden index and demonstrating a sensitivity of 54.5%, a specificity of 99.2%, a positive predictive value of 85.7%, and a negative predictive value of 95.9% for the development of complications.

## 4. Discussion

Maternal and newborn outcomes in women diagnosed with preeclampsia with severe features are significantly influenced by early detection and treatment. The challenges are exacerbated in developing countries, such as Mexico, where outcomes are less favorable, irrespective of gestational age or clinical severity. While numerous risk factors associated with adverse maternal and fetal outcomes are recognized, quantifying and predicting these risks remain challenging, owing to a lack of comprehensive understanding of the interdependence of various risk factors [10].

Efforts to identify women at risk of complications from preeclampsia with severe features are crucial to minimizing unnecessary and potentially harmful medical interventions. In 2011, the PIERS (Preeclampsia Integrated Estimate of RiSk) score was introduced to assess maternal vital signs, symptoms, and laboratory results to create a reliable algorithm predicting maternal and perinatal outcomes in women with preeclampsia [11]. However, the full-PIERS model's applicability, especially in primary care settings of low- and middle-income countries like Mexico, was hindered by the inclusion of extensive laboratory tests and validation limitations in populations like the one studied.

Our study, encompassing 130 pregnant women with a median age of 27 years, revealed that 8.5% experienced complications. Those who faced complications were characterized by specific demographic and clinical factors, including lower parity, lower history of caesarean section, higher levels of ALT and creatinine levels, and lower corticoid use. Complications were also associated with a higher fetal death rate, though other perinatal or neonatal outcomes did not significantly differ.

Our analysis demonstrated that the full-PIERS score has a good diagnostic performance, with an acceptable ROC curve of 0.718. After identifying the cut-off point that weights the best performance with the most balanced sensitivity and specificity, we documented that a cut-off point of 7.95% was associated with a sensitivity of 54.5% and specificity of 99.2% for predicting complications. Our findings suggested a higher specificity than sensitivity, indicating the model's proficiency in identifying women unlikely to develop complications. The global full-PIERS score found in our study was 0.5% (Table 3), considering that our research encompassed patients with early-onset preeclampsia with severe features, this may be regarded as low. Nonetheless, analyzing the data, most of the patients did not have alterations in laboratory parameters and were diagnosed with a systolic pressure ≥160 mmHg or diastolic pressure ≥110 mmHg on two occasions more than 15 minutes apart (Table 2), explaining our low global full-PIERS score.

Comparison with other studies underscored variations in predictive capabilities. Sharma et al. conducted a study to determine the predicted percentage probability of complications in women with preeclampsia using the full-PIERS model within the first 24 hours after admission and evaluating the model's predictive value for preeclampsia complications. The incidence of complications in their patients was higher than in our study, with 39.5% developing maternal complications (46.9% fetal complications and 62.1% experiencing both). They reported an area under the ROC curve of 0.843, which was greater than our sample, demonstrating its good discriminatory capacity to predict complications between 48 hours and 7 days after admission. The sensitivity and specificity of the model with a cut-off value ≥ 5.9% for predicting adverse maternal outcomes were 60% and 97%, respectively; and for predicting combined fetomaternal complications with a cut-off value of 4.9%, they were 44% and 96%, respectively [12]. The cut-off was lower than that reported by us, where we found that the best cut-off point, with better performance, was 7.95%. However, as we found, the full-PIERS score seems to have better specificity than sensitivity for patient identification, meaning it is more effective at identifying women who will not develop complications than those who will, presenting a high cut-off point.

Oliveira et al. observed in their Brazilian patients that within the lowest PIERS risk range (with a percentage of 1% or less), women in the cohort had a shorter interval between admission and delivery and underwent more caesarean deliveries compared to the full-PIERS cohort. Their results revealed differences in the management of preeclampsia between the validated cohorts, with low-risk women delivering earlier and more frequently by caesarean section in Brazil [13]. These distinctions suggest the necessity for a shift in obstetric management to reduce the high incidence of preterm births and caesarean sections among women with preeclampsia. Using the full-PIERS as an assessment tool could aid in avoiding unnecessary preterm births due to preeclampsia.

Bose and Wagh analyzed the data associated with the full-PIERS and mini-PIERS models and found that the full-PIERS and mini-PIERS had an overall diagnostic accuracy of 90% and 91%, respectively [14], demonstrating their good discriminatory capacity. Cazares-Avalos conducted a study similar to ours on women from Sonora, Mexico. They concluded that the full-PIERS model had a sensitivity of 58.3% and a specificity of 95.5%, with an AUC of 0.799 [5]. This result indicates a similarly strong discriminatory capacity.

Almeida et al. conducted a study to validate the full-PIERS model, measuring its ability to predict complications in pregnant women with preeclampsia in Brazil. They identified an AUC of 0.72, establishing a cut-off point of 1.7%, which is considerably lower than in our study. In the multivariate analysis they performed, no significant results were obtained [15]. With this, the authors demonstrate the discriminatory validity of the scale for their population, using a much lower cut-off point than reported in other literature, including ours.

While our study demonstrated good diagnostic performance, the limited data on women with complications pose a challenge. The small cohort may have influenced the high cut-off point and low sensitivity relative to specificity. Future research with a larger and more diverse cohort is recommended to enhance the validity of our conclusions.

## 5. Conclusion

In conclusion, our study contributes valuable insights into the diagnostic performance of the full-PIERS score, showcasing its potential as a predictive tool for complications in preeclampsia with severe features. The identified cut-off point of 7.95% provides a balanced sensitivity and specificity, offering a practical guide for clinical application.

## Figures and Tables

**Table 1:** Analysis of the demographic characteristics, obstetric-gynecological history, and prenatal care.

Variable	Global	Complications		*P*
		Yes	No	
*Demographic characteristics*				
Age (years)	27 (21–34)	27 (22–32)	27 (21–34)	0.847
Maternal weight (kg)	80 (69–90)	80 (60–90)	80 (69–90)	0.648
Maternal height (m)	1.58 (1.54–1.60)	1.58 (1.55–1.60)	1.58 (1.54–1.60)	0.98
BMI (kg/m^2^)	32.3 (27.7–37.2)	32 (25–35.2)	32.3 (27.8–37.5)	0.625
Smoking	15 (11.5%)	4 (36.4%)	11 (9.2%)	0.024

Civil status				0.001
Free union	93 (71.5%)	4 (36.4%)	89 (74.8%)	
Married	26 (20%)	7 (63.6%)	19 (16%)	
Single	11 (8.5%)	0 (0%)	11 (9.2%)	

Scholarship				0.956
Illiterate	1 (0.8%)	0 (0%)	1 (0.8%)	
Primary	22 (16.9%)	2 (18.2%)	20 (16.8%)	
Secondary	70 (53.8%)	5 (45.5%)	65 (54.6%)	
High school	30 (23.1%)	3 (27.3%)	27 (22.7%)	
Degree	7 (5.4%)	1 (9.1%)	6 (5%)	

Occupation				0.488
Home	125 (96.2%)	11 (100%)	114 (95.8%)	
Employee	5 (3.8%)	4 (36.4%)	11 (9.2%)	

*Obstetric-gynecological history and prenatal care*				

Prenatal care	4 (2–6)	3 (3–5)	4 (2–7)	0.68
Adequate prenatal care	83 (63.8%)	9 (81.8%)	74 (62.2%)	0.195
Parity	2 (1–3)	1 (1–2)	2 (1–3)	0.011

Categorical parity				0.12
Multigravida	86 (66.2%)	5 (45.5%)	81 (68.1%)	
Nulligravida	44 (33.8%)	6 (54.5%)	38 (31.9%)	
History of vaginal birth	47 (36.1%)	5 (45.5%)	42 (35.3%)	0.358
History of caesarean section	52 (40%)	1 (9.1%)	51 (42.9%)	0.025
History of miscarriage	23 (17.7%)	0 (0%)	23 (19.3%)	0.107
Weeks of gestation	31.5 (30–33)	31.6 (28–33.2)	31.5 (30–33)	0.823

**Table 2:** Comparison of the clinical presentation of women who did or did not present complications associated with preeclampsia.

Variable	Global	Yes	No	*P*
*Complications*				
Dyspnea	0 (0%)	0 (0%)	0 (0%)	—
Chest pain	3 (2.3%)	1 (9.1%)	2 (1.7%)	0.235
Oxygen saturation (%)	98 (97–99)	98 (97–98)	98 (97–99)	0.143
Platelets (K/mm3)	217 (154–272)	276 (79–298)	216 (158–266)	0.509
Creatinine (mg/dL)	0.6 (0.5–0.72)	0.73 (0.63–1.02)	0.6 (0.49–0.7)	0.005
AST (U/L)	22 (13–31)	28 (18–181)	21 (15–32)	0.061
ALT (U/L)	18 (13–31)	25 (17–105)	18 (12–29)	0.03
Systolic blood pressure (mmHg)	165 (160–175)	160 (160–172)	166 (160–176)	0.422
Diastolic blood pressure (mmHg)	110 (100–110)	110 (100–110)	110 (100–110)	0.841
Proteins (mgdL)	899 (396–3441.4)	2712 (384–7804)	846.5 (399.7–3140)	0.129

**Table 3:** Full-PIERS predictor score, medical management received, and perinatal and neonatal outcomes associated with the development of complications.

Variable	Global	Complications		*P*
		Yes	No	
*Full-PIERS predictor score and medical management received*				
Full-PIERS	0.5 (0.3–1.5)	8.7 (0.3–25.7)	0.5 (0.2–1.3)	0.017
Magnesium sulfate administration	119 (100%)	11 (100%)	119 (100%)	—
Corticosteroids	105 (80.8%)	4 (36.4%)	101 (84.9%)	0.001
Antihypertensives	128 (98.5%)	11 (100%)	117 (98.3%)	0.837

*Perinatal and neonatal outcomes*				
Days of hospital stay	5 (4–6)	5 (4–6)	5 (4–6)	0.723
Admission-to-delivery interval, days	2 (1–2)	1 (1–2)	2 (1–2)	0.156

Perinatal outcome				0.036
Alive	126 (96.9%)	9 (81.8%)	117 (98.3%)	
Death	4 (3.1%)	2 (18.2%)	2 (1.7%)	
Newborn weight (g)	1620 (1250–2030)	1670 (1110–2310)	1620 (1250–2022)	0.8

Type of delivery				0.302
Caesarean section	117 (90%)	9 (81.8%)	108 (90.8%)	
Birth	13 (10%)	2 (18.2%)	11 (9.2%)	

**Table 4:** Cut-off points obtained from full-PIERS.

full-PIERS cut	Sensitivity (%)	Specificity (%)	Youden index
1.05	63.6	69.7	0.333
2.05	54.5	81.5	0.36
3.05	54.5	87.4	0.419
4.05	54.5	94.1	0.486
5	54.5	96.6	0.511
6.75	54.5	98.3	0.528
7.95^*∗*^	54.5	**99.2**	**0.537**
8.75	45.5	99.2	0.447
11.55	45.5	100.0	0.455
18	36.4	100.0	0.364

The bold value in Table highlights the cutoff point with the best diagnostic performance, the highest Youden index (0.537), and a sensitivity of 54.5% and specificity of 99.2% for complications.

## Data Availability

The data used to support this study's findings are restricted by the Ethics Committee of the Hospital Regional Materno Infantil del Estado de Nuevo León to protect patients' privacy. Data are available from Dr. Eduardo Ponce Nájera through mail eponcenajera@gmail.com, for researchers who meet the criteria for access to confidential data.
